# Prevalence of obesity, diabetes and hypertension in immigrant populations in northeastern Mexico

**DOI:** 10.3389/fpubh.2023.1220753

**Published:** 2024-01-11

**Authors:** Guillermo Baudelio Gómez-Morales, Brenda Sofía Rosas-Torres, Williams Jesús Hernández-Jiménez, Estefanía Mattenberger-Cantú, Javier Vargas-Villarreal, Horacio Almanza-Reyes, Francisco González-Salazar

**Affiliations:** ^1^Northeast Biomedical Research Center, Institute of Mexican Social Security, Monterrey, Mexico; ^2^Basic Science Department, University of Monterrey, Garza García, Mexico; ^3^Faculty of Medicine and Psychology of the Autonomous, University of Baja California, Tijuana, Mexico

**Keywords:** Central American immigrant, obesity, diabetes, hypertension, Central American immigrants in Mexico

## Abstract

**Introduction:**

Hispanic immigrants are a fast-growing population in the United States of America (USA) that disproportionately suffer from chronic diseases. Despite the increasing prevalence of obesity in Latin-American countries, only a few studies have examined the onset of chronic diseases in Mexican and Central American migrants in Mexico.

**Objective:**

The objective of this study is to determine the prevalence of obesity, diabetes, and hypertension in Central American immigrants who are in the process of traveling through northeastern Mexico to the United States.

**Methods:**

An observational, descriptive, cross-sectional study was conducted among migrants, mostly Central Americans. Migrants who agreed to participate in the study were interviewed face-to-face by researchers to obtain their sociodemographic data. To obtain the prevalence, many health indicators related to obesity, diabetes, and hypertension, including weight, height, fasting glucose, and blood pressure, were measured.

**Results:**

In total, 520 migrants were interviewed; sociodemographic data indicated that most participants were men (76%), from Honduras (72.6%), single (61.2%), and have elementary level of education (48.6%). The somatometric evaluation revealed that 28.9% were diagnosed as overweight, 10.7% with obesity, and 3.3% with malnutrition. Of less prevalence, 8.8% were detected with hypertension and 4.6% had fasting hyperglycemia. The mean participant age was 29.11 ± 10.00 years. For each participant, the average weight was 66.72 ± 13.09 kg; the average height was 1.64 ± 0.08 m; the average body mass index (BMI) was 24.59 ± 4.32; the mean systolic and diastolic pressures were 116.26 ± 15.13 and 74 ± 9.65, respectively; and the average glycemia was 100.97 ± 21.99. El Salvador showed the highest proportion of people with diabetes (14.7%). Women who participated in this study had a higher proportion of obesity (23.4%, *p* = 0.02) and overweight (36.2%) than men (8.4 and 29.2%, respectively). People from Mexico, Nicaragua, and Honduras reported a high prevalence of overweight participants (63.6, 47.4, and 30.7%, respectively), while people from El Salvador and Nicaragua had a high prevalence of obese participants (23.5 and 21.1%, respectively).

**Conclusion:**

We found significant differences in the rates of obesity, diabetes, and hypertension between groups of Central American migrants and their place of origin, age, educational level, and gender. Our findings highlight the importance of exploring differences within groups of Central American migrants traveling through northeastern Mexico to the United States, which may explain several health indicators.

## 1 Introduction

Obesity is a worldwide epidemic caused by an increase in the consumption of foods with poor nutritional value, rich in sugars with high fructose content, and with saturated trans fats. This, accompanied by a lack of exercise, causes the accumulation of fat in tissues (1). The United States and Mexico have high rates of obesity (2). However, Central American countries are not exempt from suffering from eating disorders due to bad eating habits that cause an imbalance between energy intake and expenditure (3–6). Obesity is often accompanied by significant comorbidities such as diabetes and hypertension (7, 8). It is important to identify these disorders early because when diabetes and hypertension become complicated, they can be extremely disabling and may lead to significant economic losses in the health sector due to long-term treatments. In Mexico, the prevalence of diabetes has reached 14% of the adult population and is one of the leading causes of mortality, representing 14% of all deaths in 1 year (9). Hispanic immigrants are a fast-growing population in the United States. According to data from the United States census (10), the Latin population reached a total of 62.5 million people in 2021, which shows a 19% increase from the 50.5 million of Latinos in 2010. Therefore, there is a high prevalence of general obesity among Latin immigrants (11).

Immigrants are characterized by being young people who regularly travel alone and who would ideally be healthy and without any chronic-degenerative diseases (12). However, with increasing frequency, immigration populations are shifting to include caravans or groups where entire families travel to the United States (13). There are reports indicating that Latino, Mexican, and Central American migrants change their eating habits and suffer from obesity while living in the United States for years (14). To date, the presence of obesity, diabetes, and hypertension has not been characterized in the population of Central American migrants while transiting through the Mexican territory. Most of the studies are of Central American migrants in the United States (15). Thus, the main objective of this research was to determine the prevalence of obesity, diabetes, and hypertension in Central American immigrants in the process of traveling through northeastern Mexico to the United States. Comparing the prevalence of these conditions in the country of transit (northeastern Mexico) with those reported in the countries of destinations (United States) and ejection (Central American countries) will guide in decision-making of public policies and programs to achieve significant effectiveness and efficiency in the administration of resources to prevent diabetes, obesity, and hypertension across all the regions.

## 2 Materials and methods

An observational, descriptive, cross-sectional study was carried out in a migrant shelter called Casa Nicolás. This shelter is located in Guadalupe Nuevo Leon, a metropolitan zone of Monterrey Mexico, and hosts approximately 1,500 migrants per year. Guadalupe city is a town with a population of 643,143 habitants. This investigation was carried out from January 2019 to March 2021. During this period, the majority of the people hosted in this shelter were Central Americans, mainly from Honduras, Guatemala, Nicaragua, and El Salvador.

### 2.1 Sample and sample size

Considering a population of 1,500 migrants in 1 year, a sample size was calculated using Fisterra’s online sample size calculator. With a confidence level of 95%, we looked for a sample size sufficient to detect a proportion of diabetes of 4% with an accuracy of 2%. In this way, the calculated sample size was 369 migrants, with an adjustment to 434 individuals consisting of up to 15% of the individuals who did not want to perform all the tests. The sampling was non-probabilistic by quota. The data were collected in the shelter every week during the year 2019 until March 2021 to complete the desired sample size.

### 2.2 Selection criteria

Migrants over the age of 13 years who spent the night in the shelter were asked for their signed written consent to participate in the study. People over 18 years of age signed a consent, while those under 18 years of age were asked for their signed assent along with parental consent. Migrants older than 13 years of any gender, nationality, or religion who gave consent to participate were included in this study. Migrants who stated that they had left their country of origin for more than 6 months or who commented that they had lived in Monterrey, Mexico for a longer time were excluded. This is because after spending an average of 6 months living in Guadalupe, Nuevo León, they adapt to the food and customs that are very different from those of their country of origin. People whose surveys were illegible were removed from the study.

### 2.3 Procedures

The people who agreed to participate were interviewed early in the morning to collect their sociodemographic data. After completing the interview, the following procedures shown in Sections 3.4–3.8 were performed.

### 2.4 Weight and height

With the least amount of clothing possible, on an empty stomach, patients were weighed on a Nuevo León S.A. DE C.V., brand pedestal scale. The scale model was P-220, and the material was iron, with measurements of 26.8 cm × 36.8 cm, with a capacity of 220 kg. The manufacturing place is Monterrey, Mexico. Weight was recorded in kilograms with a precision of one decimal.

### 2.5 Height

On a pedestal scale, with their back facing the wall, height was measured using the stadiometer of the scale brand Nuevo León S.A. DE C.V. Height was recorded in meters.

### 2.6 Glucose

Fasting blood glucose was determined using a Johnson and Johnson brand glucometer, model OneTouch Ultra TM 2. After following a hand hygiene routine and cleaning with a 70% isopropyl alcohol swab, a finger was punctured with a lancet and pressed in the same manner to extract a drop of blood. This drop of blood was placed on a Johnson and Johnson glucose test strip, model OneTouch Ultra. The reading from the device was manually transcribed with a pencil or pen onto the data collection sheet designed for this purpose. When fasting glucose value was greater than 126 mg/Dl, the patient was considered to have diabetes mellitus (DM), whereas the patient was considered without DM when the reading was 125 mg/dL or less.

### 2.7 Blood pressure

Blood pressure was taken in all individuals who accepted the procedure. The conditions for measuring blood pressure were as follows: migrants had to be seated and at rest (participants were asked to make inhalations and exhalations repeatedly to be completely relaxed) for at least 15 min before taking the blood pressure sample using a Microlife model BP3UG1-2E automatic blood pressure measuring cuff. The cuff was placed on the patient’s left arm, two finger widths above the elbow crease, and adjusted so that it was not too tight or too loose on the arm. The cuff was properly aligned with the brachial artery, and the start button of the device was pressed to begin measurement. The systolic and diastolic blood pressure readings were recorded. Data were manually collected with a pen on the corresponding data collection sheet. For the registry, individuals were considered to have arterial hypertension (HBP) when the systolic pressure was greater than or equal to 140 mmHg and the diastolic pressure was greater than or equal to 90 mmHg. Individuals were considered to be without HBP when the systolic pressure value was less than 140 mmHg and the diastolic pressure was less than 90 mmHg.

### 2.8 Somatometric diagnosis

To carry out the somatometric diagnosis of the individuals, the formula of weight in kg/ $\mathrm{height}\;\mathrm{in}\;m^{2}$ = body mass index (BMI) was used. The result of this formula was compared with internationally valid somatometric diagnostic tables by the World Health Organization (WHO) (16). The BMI figures less than 18.5 m^2^SC indicate underweight, as shown in Table 1 as malnutrition. The figures between 18.5 m^2^SC and 24.9 m^2^SC indicate normal weight. The figures between 25 m^2^SC and 29.9 m^2^SC indicate overweight, and the values greater than or equal to 30 m^2^SC indicate obesity.

**Table 1:** Somatometric diagnosis, diabetes, and hypertension.

Variable	Outcome	*n*	Frequency	Percentage
Somatometric diagnosis	Normal	513	293	57.1
	Overweight		148	28.9
	Obesity		55	10.7
	Malnutrition		17	3.3
Detected hypertension	No	512	467	91.2
	Yes		45	8.8
Detected hyperglycemia	No	470	448	95.4
	Yes		22	4.6

The numbers of participants are *n*; the percentage is %.

### 2.9 Statistical analysis

The data of the people previously stored in a Microsoft Excel^®^ database were analyzed with the help of the IBM^®^ SPSS^®^ Statistics V25 program. All quantitative data were used to calculate means and standard deviations, while qualitative data were used to calculate proportions. The proportion of obesity, overweight, diabetes, and hypertension was calculated by dividing the number of people with the characteristic over the total number of individuals evaluated with this procedure. Finally, the associations between the presence of obesity, overweight, diabetes, and hypertension were calculated, contrasting with the proportions of the rest of the independent variables, for which the chi-square statistic was used. Differences between proportions were considered statistically significant when value of ps were less than ≤0.05.

### 2.10 Ethical considerations

To carry out this research, the protocol was first registered in the ethics and research committee of the University of Monterrey, and it was registered with the number 042016-CIE. Authorization was then requested from the shelter authorities in which they agreed to the weekly survey of the migrants since their population changes every 3 days due to the shelter’s stay regulations. All adult participants signed an informed consent, while the minors signed an assent document and their parents signed the informed consent. During the course of this project, international laws for conducting research studies with humans were respected, including the Helsinki Code and the general health law of the United Mexican States. Patient data were kept confidential at all times.

## 3 Results

### 3.1 Sociodemographic characteristics of the study

For the present study, there were a total of 520 participants (Table 2). According to sociodemographic data, the majority of participants (76%) were men, and they mainly came from Honduras (72.6%). Most of the participants were single (61.2%) and had elementary level of education (48.6%). Most of the immigrants (66.1%) traveled alone, 31.2% traveled with family, and ony 2.8% traveled with friends. The mean age of the migrants traveling from El Salvador was 27.8 ± 7.38 years, while the mean age of migrants traveling from Nicaragua was 39.0 ± 12.15 years, from Guatemala was 31.2 ± 9.86 years, and from Mexico was 30.0 ± 11.51 years.

**Table 2:** Sociodemographic characteristics.

Variable	Outcome	Frequency	Percentage
Gender	Men	395	76.0
	Women	105	20.2
	Did not answer	20	3.8
Country of origin	Honduras	381	72.6
	Salvador	36	6.5
	Guatemala	34	6.2
	Nicaragua	22	4.0
	Did not answer	20	3.6
	Mexico	19	3.4
	Cuba	3	0.5
	Costa Rica	3	0.4
	Ecuador	2	0.2
Marital status	Single	320	61.2
	Free union	94	17.6
	Married	80	14.5
	Unknown	19	3.4
	Separated	3	0.4
	Widower	2	0.2
	Engaged	2	0.2
Education	Elementary school	257	48.6
	Middle school	112	21.4
	High school	68	12.3
	Did not answer	35	6.4
	None	30	5.4
	Degree	18	3.3
Travel alone	Yes	348	66.1
	No	172	32.9
Travel with family	Yes	162	31.2
	No	358	68.8
Travel with friends	Yes	15	2.8
	No	505	97.2
Travel status	Travel alone	345	66.3
	Not travel alone	175	33.7

The numbers of participants are n; the percentage is %.

### 3.2 Comorbidities in the study

Regarding previous comorbidities diagnosed, it was found that, among the people interviewed, 3.2% referred to have a previous diagnosis of diabetes and 4.5% of hypertension; however, only 0.7% of the people were suffering from psychiatric/mental illness. Additionally, 88.1% consumed alcoholic beverages, whereas 29.8% mentioned smoking (Table 3).

**Table 3:** Comorbidities according to survey results.

Variable	Outcome	Frequency	Percentage
Diabetes	No	437	96.8
	Yes	14	3.2
Hypertension	No	431	95.5
	Yes	20	4.5
Mental alterations	No	448	99.3
	Yes	3	0.7
Smoke	No	317	70.2
	Yes	134	29.8
Consume alcoholic drinks	No	54	11.9
	Yes	397	88.1

Percentage is %.

### 3.3 Somatometric diagnosis, diabetes, and hypertension of the study

Regarding the somatometric diagnosis (reported in Table 1), 28.9% were diagnosed as overweight, 10.7% with obesity, and 3.3% with malnutrition; this adds up to 42.9% of people who have somatometric issues. In addition, only 8.8% of people were detected with hypertension, while 4.6% had fasting hyperglycemia. As shown in Table 4, for each participant, the average age was 29.11 ± 10.00 years; the average weight and height were 66.72 ± 13.09 kg and 1.64 ± 0.08 m, respectively; the average BMI was 24.59 ± 4.32 kg/m^2^; the mean systolic and diastolic pressures were 116.26 ± 15.13 mmHg and 74 ± 9.65 mmHg, respectively; and the average glycemia was 100.97 ± 21.99 mg/dL. Table 5 shows the cross-tabulation of sociodemographic characteristics and diabetes. In terms of gender, it was found that men had hyperglycemia more often than women, but it was not statistically significant. Additionally, people with a married status and those who traveled alone had a higher proportion of diabetes, and those with the highest proportion of diabetes had a higher level of education, but none of these differences were statistically significant. However, it was found to be statistically significant (*p* = 0.02) that El Salvador had the highest proportion of people with diabetes (14.7%) compared to migrants in other countries such as Guatemala or Mexico where no cases of diabetes were reported.

**Table 4:** Relationship between anthropometric characteristics, hypertension, and diabetes.

Variable	*n*	Range	Minimum	Maximum	Half	Deviation
Age (in years)	520	62	13	62	29.11	10.00
<20	106	20	13	20	17.74	1.61
21–40	337	40	21	40	28.92	4.66
41–59	75	59	41	59	46.25	3.52
>60	2	62	60	62	61	1
Weight	513	85	28	113	66.72	13.09
Height	516	0.720	1.20	1.92	1.64	0.18
BMI	513	30.44	14.28	44.73	24.59	4.32
Systolic pressure	512	169	110	180	116.26	15.13
Diastolic pressure	512	70	45	115	74.13	9.65
Glycemia	470	279	63	342	100.97	21.99
N valid (per list)	451					

The numbers of participants are *n*.

**Table 5:** Crossed table diabetes with sociodemographic characteristics.

Variable	Outcome	Without hyperglycemia	With hyperglycemia	*p*-value
Gender	Men	316 (95.2)	16 (4.8)	0.53
	Women	90 (95.7)	4 (4.3)	
Country of origin	Honduras	316 (96)	13 (4.0)	0.02*
	Guatemala	33 (100)	0 (0.0)	
	Salvador	29 (85.3)	5 (14.7)	
	Nicaragua	17 (89.5)	2 (10.5)	
	Mexico	11 (100)	0 (0.0)	
Marital status	Single	266 (96.0)	11 (4.0)	0.23
	Married	140 (94.0)	9 (6.0)	
Education	Middle school or less	321 (95.3)	16 (4.7)	0.64
	High school or less	69 (94.5)	4 (5.5)	
	Did not answer	16 (100.0)	0 (0.0)	
Travel alone	No	134 (97.1)	4 (2.9)	0.16
	Yes	272 (94.4)	16 (5.6)	
Travel with family	No	282 (94.6)	16 (5.4)	0.23
	Yes	124 (96.9)	4 (3.1)	

Frequencies above reflect percentages among those providing data on the corresponding variable. *p ≤ 0.05.

### 3.4 Relationship of diabetes and different comorbidities in the study

The cross-tabulation of some comorbidities of the participants and the presence or absence of diabetes is shown in Table 6. Regarding the presence of hypertension, it is important to note that there was a higher proportion (23.1) of people with hypertension and diabetes, with a statistical significance of 0.005. Furthermore, it is clear that hypertension is the variable that gives a differentiated diagnosis of diabetes. As shown in Table 6, hypertension is the only statistically representative comorbidity in our population compared to smoking, alcohol consumption, and somatometric diagnosis relationship. Participants with a BMI indicating malnutrition, obesity, and overweight have a higher proportion of diabetes (in that order) compared to those with a normal BMI, but these differences were not statistically significant.

**Table 6:** Relationship of diabetes and different comorbidities.

Variable	Outcome	Without hyperglycemia	With hyperglycemia	*p*-value
Hypertension	No	318 (95.5)	15 (4.5)	0.005*
	Yes	10 (76.9)	3 (23.1)	
Smoke	No	225 (94.5)	13 (5.5)	0.55
	Yes	103 (95.4)	5 (4.6)	
Consume alcoholic drinks	No	288 (94.7)	16 (5.3)	0.58
	Yes	40 (95.3)	2 (4.8)	
Somatometric diagnosis	Normal	229 (97.4)	6 (2.6)	0.13
	Overweight	122 (93.1)	9 (6.9)	
	Obesity	46 (92.0)	4 (8.0)	
	Malnutrition	9 (90.0)	1 (10.0)	

Frequencies above reflect percentages among those providing data on the corresponding variable. *p ≤ 0.05.

### 3.5 Somatometric diagnosis with sociodemographic characteristics of the study

Table 7 shows that, in the group including participants with malnutrition, obesity, and overweight, women have a higher proportion of obesity (23.4%) and overweight (36.2%) than men (8.4 and 29.2%, respectively), with statistically significant differences. People from Mexico, Nicaragua, and Honduras were more overweight (63.6, 47.4, and 30.7%, respectively), and people from El Salvador and Nicaragua were more obese (23.5 and 21.1%, respectively). Married people were more overweight and obese than those who were single (34.2 and 20.1%, respectively). People who travel with family were more overweight and obese than those who travel alone (34.4 and 22.7%, respectively). Finally, Table 8 indicates that overweight, obesity, and hypertension were much more common in people who presented with diabetes during the fasting blood glucose test. Additionally, people who smoke or drink alcoholic beverages were more likely to be obese.

**Table 7:** Crossed-table somatometric diagnosis with sociodemographic characteristics.

Variable	Outcome	Normal	Overweight	Obesity	Malnutrition	*p*-value
Gender	Men	199 (59.9)	97 (29.2)	28 (8.4)	8 (2.4)	0.53
	Women	36 (38.3)	34 (36.2)	22 (23.4)	2 (2.1)	
Country of origin	Honduras	189 (57.4)	101 (30.7)	32 (9.7)	7 (2.1)	0.02*
	Guatemala	19 (57.6)	8 (24.2)	6 (18.2)	0 (0.0)	
	Salvador	17 (50.0)	6 (17.6)	8 (23.5)	3 (8.8)	
	Nicaragua	6 (31.6)	9 (47.4)	4 (21.1)	0 (0.0)	
	Mexico	4 (36.4)	7 (63.6)	0 (0.0)	0 (0.0)	
Marital status	Single	167 (60.3)	80 (28.9)	20 (7.2)	10 (3.6)	0.23
	Married	68 (45.6)	51 (34.2)	30 (20.1)	0 (0.0)	
Education	Middle school or less	185 (54.9)	102 (30.3)	41 (12.2)	9 (2.7)	0.64
	High school or more	39 (53.4)	25 (34.2)	8 (11.0)	1 (1.4)	
	Not answered	11 (68.8)	4 (25.0)	1 (6.3)	0 (0.0)	
Travel alone	No	60 (43.5)	45 (32.6)	29 (21.0)	4 (2.9)	0.16
	Yes	175 (60.8)	86 (29.9)	21 (7.3)	6 (2.1)	
Travel with family	No	184 (61.7)	87 (29.2)	21 (7.0)	8 (2.0)	0.23
	Yes	51 (39.8)	44 (34.4)	29 (22.7)	4 (3.1)	

Frequencies above reflect percentages among those providing data on the corresponding variable. *p ≤ 0.05.

**Table 8:** Comorbidities with somatometric diagnosis.

Variable	Outcome	Normal	Overweight	Obesity	Malnutrition	*p*-value
Diabetes	No	200 (59.2)	101 (29.9)	30 (8.9)	7 (2.1)	0.002*
	Yes	2 (25.0)	5 (62.5)	1 (12.5)	0 (0.0)	
Hypertension	No	197 (59.2)	99 (29.7)	30 (9.0)	7 (2.1)	0.003*
	Yes	5 (38.5)	7 (53.8)	1 (7.7)	0 (0.0)	
Smoke	No	132 (55.5)	76 (31.9)	26 (10.9)	4 (1.7)	0.002*
	Yes	70 (64.8)	30 (27.8)	5 (4.6)	3 (2.8)	
Consume alcoholic drinks	No	173 (56.9)	95 (31.3)	30 (9.9)	6 (2.0)	0.003*
	Yes	29 (69.0)	11 (26.2)	1 (2.4)	1 (2.4)	

Frequencies above reflect percentages among those providing data on the corresponding variable. *p ≤ 0.05.

## 4 Discussion

This study shows statistically significant differences in the prevalence of obesity, diabetes, and hypertension according to the country of origin for Mexican and Central American migrants from a migrant shelter in Guadalupe, Nuevo Leon. The estimated cost of diabetes mellitus in 2000 was $ 65 billion in Latin America and the Caribbean (17). This burden was attributed to lost productivity due to mortality and disability, as well as direct medical costs caused by the treatment of diabetes and its long-term complications (17). Diabetes, cardiovascular diseases, and obesity represented economic losses in the United States for more than 2.8 billion dollars annually, which represents 7% of the total health expenditure. In Mexico, health expenses for diabetes and its complications were more than $1.2 billion in 2023 (18). No studies examining the costs of diabetes or other chronic diseases have been published in Honduras. However, there is only one study from the Coordination of Chronic Noncommunicable Diseases of the Ministry of Health where the estimated cost of diabetes mellitus in 2015 was 1.29% from the gross domestic product (GDP), which was expected to increase to 2.59% in 2020 (19). Barcelo et al. (20) conducted a study to determine the direct costs (healthcare expenses such as insulin, oral hypoglycemic agents, tests, consultations, hospitalizations, emergency visits, and complications treatment) and indirect costs (lost resources due to premature mortality and temporary and permanent disabilities) related to diabetes in 29 countries from Latin America and the Caribbean in 2015 (20). The healthcare costs of diabetes in 2015 were annually estimated to be $1,039 billion in Honduras, $1,471 billion in Guatemala, $628 million in Nicaragua, $2,545 billion in Cuba, $814 million in Costa Rica, and $2,537 billion in Ecuador (20). The financial consequences of the increase of diabetes in countries considered as expellers represent one of the main challenges to health systems, indicating a need to implement public policies and programs to achieve greater effectiveness and efficiency in controlling, diagnosing, and treating diabetes in migrants from countries in transit and in receptor countries.

For this reason, the evidence of the prevalence of obesity, diabetes, and hypertension in the countries of origin in comparison with the country of transit present could explain the differences. However, according to data from previously reported publications, the prevalence of diabetes in the countries of origin does not align with the differences that this investigation found. For example, according to the STEP survey carried out in Guatemala in 2015 (21), it was reported that 5.7% of the population reported having DM in the age range of 18–44 years, contrary to what our results report, which has a prevalence of 0%. In Mexico, data from the 2018 national health survey reported a prevalence of 1.8% in the age group of 20–39 years (22), whereas data from our survey showed that migrants from Mexico reported a prevalence of 0%. These differences in the percentage of diabetes in the group of migrants from Guatemala and Mexico may be due to two factors. First, there was a small number of participants from both countries (34 participants from Guatemala and 19 from Mexico), the majority fit into a relatively young group (age between 18 and 44 years), and none were diabetic. Second, various investigations show that immigrants have a significantly lower daily intake of sugary drinks, processed red meats, and sodium. They also have a higher intake of vegetables, fruits, and whole grains compared to people born in the United States (23, 24). These findings support the idea that immigrants have more favorable health behaviors that are associated with a lower risk of chronic diseases (23, 24). Interestingly, the data from El Salvador obtained in our study indicated a prevalence of diabetes of 14.7% in people with an average age of 31 years. These data contrast with the results of the National Survey of Chronic Noncommunicable Diseases in the Adult Population of El Salvador, which reported a 3.2% prevalence of diabetes mellitus in the age group of 20–40 years (25). These findings in the migrant group from El Salvador could be attributed to genetic and behavioral differences, which make different ethnic subgroups more susceptible to diabetes rather than hypertension (26). Previous studies indicate that immigrants have differences in lifestyle and diet that may affect their risk of developing chronic diseases such as hypertension (27, 28). It is well known that more recent immigrants have lower blood pressure than those who live in the United States for longer periods of time (29, 30). Data from our study found that people traveling from Nicaragua with an average age of 39 years had a 10.5% prevalence of diabetes. These data contrast greatly with the results obtained in the Survey of Diabetes, Hypertension and Risk Factors for Chronic Diseases in Nicaragua, which indicated a prevalence of 2.0% in participants within the age range of 20–39 years (31). Additionally, a study conducted by the National Autonomous University of Nicaragua found that, in the north, center, and Pacific of Nicaragua, there was a 4.6% prevalence of diabetes mellitus in people between 30 and 39 years of age (32). Finally, from the data of 326 people from Honduras with an average age of 28 years, it was found that the prevalence of diabetes was 4.0%. This finding is very similar to the data reported by Bermúdez-Madriz et al. (33) in the review article about the health system in Honduras, reporting a 6.2% prevalence (group <19 years in urban areas) of DM (33).

In a recently published meta-analysis from (11), 52 studies with 436,654 immigrant Latinos in the United States reported a pooled prevalence for arterial hypertension of 28% (95% confidence intervals (CIs): 23–33%), type 2 diabetes mellitus of 17% (95% CIs: 14–20%), general obesity of 37% (95% CIs: 33–40%), and abdominal obesity of 54% (95% CIs: 48–59%) (11). The obtained results from our participants were as follows: The prevalence of arterial hypertension was 8.8%, type 2 diabetes mellitus was 4.6%, and general obesity was 10.7%. In previous studies, high rates of factors associated with obesity have been observed, with more than 45% of new immigrants in the United States being reported as overweight or obese. These factors vary according to the place of origin, the duration of residence in the United States and regions of current residence, and sociodemographic characteristics (15). Among the six immigrant groups studied, the group considered from Latin America and the Caribbean had higher prevalence rates of overweight and obesity than the Asian/Pacific/Oceanic and Sub-Saharan African groups. The prevalence of combined overweight and obesity was higher in men (54%) than in women (38%). Men had a higher prevalence of overweight and women had a slightly higher prevalence of obesity among immigrants from Latin America and the Caribbean, which indicates notable heterogeneity in the prevalence of overweight and obesity within and between hometown groups by age, poverty level, years of residence in the United States, and regions of current residence (15).

Regarding the previously diagnosed comorbidities, it was found that, among the people interviewed, 3.2% reported having a previous diagnosis of diabetes. Undiagnosed diabetes is an indicator of a lack of diabetes awareness. A literature review conducted by (34) reported that the prevalence of undiagnosed diabetes was higher in Honduras (range 29.9–50%), Nicaragua (43.3%), and Costa Rica (10.3–28.4%). These reported values are much higher than those determined in our study (34). This discrepancy may be because the majority of Honduran migrants have good diabetes awareness. Irazola et al. (35) described that awareness increased slightly with educational level. However, most studies do not report associations between undiagnosed diabetes and age, sex, educational level, socioeconomic status, or geographic location (34, 35). In our study, we found that migrants who had a higher prevalence of diabetes had a higher educational level; however, none of those correlations were statistically significant. In our population, 8.2% of people were detected with hypertension and 4% with diabetes. An interesting finding was that 23.1% of patients with diabetes were hypertensive, while only 4.5% of people without diabetes were hypertensive. This comorbidity has already been reported in previous studies such as the one from Petrie et al. (36), where 85% of people with diabetes also had hypertension. A review published in 2014 (37) showed that more than 50% of patients with diabetes also have hypertension. Thus, the results found in our study are consistent with what was previously reported in the literature, though the proportion is much lower. This can be explained by the sample consisting of a young population that involves a few years of evolution with diabetes and perhaps hypertension could be developed as a common comorbidity in patients with diabetes. It should be noted that Central American immigrants are a heterogeneous racial and ethnic group representing multiple countries of origin with different levels of education, socioeconomics, and cultural traditions, and these variables vary across different regions of Central America. These factors influence the prevalence of obesity, diabetes, and hypertension that has not been studied in previous research on Central American immigrants traveling through northeastern Mexico to the United States.

### 4.1 Study limitations

The first limitation of the study is that our data were gathered from a cross-sectional study of immigrants, and, therefore, the associations found are not proof of causality. Second, there is a high heterogeneity for all the results since the immigrant populations differ in many aspects, such as the country of origin (migrants from eight countries were reported), migratory status, educational levels, socioeconomic aspects, and whether they traveled accompanied or alone. These differences could explain the various inconsistencies found between the different populations of migrants. Third, we cannot definitively state whether our results are primarily due to a selection of Central American migrants bound for the United States or a selection of Central American migrants returning to Mexico. Fourth, analyses of the study’s descriptive statistics results may not be representative of all immigrants from these ethnic groups, and there may be selection bias as a result of recruitment through migrant shelter. Fifth, participation in this study was largely limited to migrants from Honduras, at 72.6% of the study population. Sixth, the measurements for blood glucose and blood pressure were only taken once in each participant, which is a limitation for monitoring their diabetes and hypertension.

### 4.2 Study strengths

Like many other studies regarding the health of immigrants, our study contains important advantages. First, the representative sample size of Central American immigrants over a 26-month period (January 2019 to March 2021) used the same sampling and data collection methodologies among its participants. Another strength of this study was its participatory approach based on the migrant community, which was guided by a multidisciplinary steering committee, comprised of academic researchers, physicians, and representatives of the migrant community, and this facilitated the appropriate study protocol and interpretations of the findings. The third strength was the use of health indicators to determine obesity, diabetes, and hypertension in the Central American migrant population. These indicators allowed us to rule out the possibility that the differences in health examinations between individuals from different Central American countries can be homogenized to have identical values in the abovementioned different countries. Fourth, our research design used data from a United States-Mexico border state, which allowed us to rule out the possibility of replicating this study in Mexican border cities with a high confluence of Central American migration.

### 4.3 Future research

First, more large-scale, multicenter studies are needed in different United States–Mexico border cities that use the same metrics to assess obesity, diabetes, and hypertension as well as facilitate the development of new interventions targeted at Central American immigrants. Second, it would be important to implement diabetes education among the Central American immigrant populations on the northern border of Mexico. Third, and most importantly, gender- and age-specific interventions must be targeted at specific immigrant groups, who are at a higher risk of developing diabetes earlier in the life course. Fourth, future research should investigate many other important socioeconomic and sociocultural factors that affect the prevalence and management of obesity, diabetes, and hypertension.

## 5 Conclusion

This study determined that diabetes does not have a homogeneous distribution in migrant populations. A notable heterogeneity was found in the prevalence of obesity, diabetes, and hypertension among the groups of Central American migrants and their place of origin, age, educational level, and gender. It should be noted that the participation in this study is largely limited to migrants from Honduras, with 72.6% of the study population. Nevertheless, with this population, it could be determined that diabetes is more common in Salvador and Nicaragua and less frequent in Honduras, Guatemala, and Mexico. The presence of increased diabetes in Salvador and Nicaragua cannot be explained by differences in the prevalence of diabetes in the countries of origin. There are no factors associated with the presence of diabetes in the population of Central American and Mexican migrants since neither age, gender, marital status, nor education were associated with a higher prevalence of diabetes. Finally, the presence of arterial hypertension as a comorbidity occurred in a lower proportion than previously reported. Obesity is most common in migrant women than men. Nicaragua and Mexico have a higher proportion of overweight participants, whereas El Salvador and Mexico have a higher proportion of obesity participants. According to the reviewed literature, this is the first study that evaluates the association between obesity, diabetes, and hypertension in a representative sample of the Central American immigrant population traveling through northeastern Mexico to the United States.

## Data availability statement

The original contributions presented in the study are included in the article/supplementary material, further inquiries can be directed to the corresponding authors.

## Ethics statement

The studies involving humans were approved by the Ethics and Research Committee of the University of Monterrey, with the number 042016-CIE. The studies were conducted in accordance with the local legislation and institutional requirements. Written informed consent for participation in this study was provided by the participants’ legal guardians/next of kin. Written informed consent was obtained from the individual(s), and minor(s)’ legal guardian/next of kin, for the publication of any potentially identifiable images or data included in this article.

## Author contributions

GG-M, BR-T, and WH-J were involved in the study design, analysis, and data collection. EM-C, JV-V, HA-R, and FG-S wrote the original manuscript, prepared the analysis, and interpreted the data. BR-T, HA-R, and FG-S helped with the analysis and gave essential comments on multiple versions. All authors approved the final version of the manuscript.
